# A dataset for predicting Supreme Court judgments in Nigeria

**DOI:** 10.1016/j.dib.2023.109483

**Published:** 2023-08-09

**Authors:** O.C. Ngige, F.Y. Ayankoya, J.A. Balogun, E. Onuiri, C. Agbonkhese, F.A. Sanusi

**Affiliations:** a Federal Institute of Industrial Research, Oshodi, Nigeria; b Department of Computer Science, Babcock University, Ilishan-Remo, Ogun State, Nigeria; c Department of Computer Science and Mathematics, Mountain Top University, Nigeria

**Keywords:** Classification algorithms, Feature extraction, Justice system, Supervised learning

## Abstract

It has been widely argued among researchers that the application of big data analytics promises to reduce human bias and provide a scientific and evidence-based approach to the judicial process. In this dataset, historical data consisting of appeal cases presented at the Supreme Court of Nigeria (SCN) were collected from an online repository (Primsol Law Pavillion). A total of 5585 appeal cases brought before the SCN were collected from the archive. The dataset consisted of both criminal and civil appeal cases brought before the SCN. Variables that are related to court case proceedings were identified from related literature, verified by legal experts and used as a basis for generating an electronic structured version of the dataset stored as a spreadsheet file from the unstructured data. From the collected data, thirteen input variables were identified with one output/decision variable. The distribution of the numerical variables was presented as a descriptive statistical summary in terms of the minimum, maximum, mode, mean and standard deviation. The developed dataset can assist researchers to build predictive systems by training their models. Various feature extraction techniques can also be applied on the dataset to remove irrelevant or redundant features for increased performance of such classifiers that are needed to predict the outcome of legal cases.

Specifications Table

**Table:** 

Subject	Computer Science
Specific subject area	Artificial Intelligence
Type of data	Table
How the data were acquired	A thorough review of the literature was done to identify suitable features found in typical court proceedings. These features were verified by highly experienced legal professionals following which data containing information about the features were collected.
Data format	Raw: csv file
Description of data collection	The dataset consisted of features extracted from both criminal and civil appeal cases of SCN. The data collected from the archive consist of case files that were stored as text-based documents in the .docx format thus presenting the data as an unstructured dataset. Furthermore, the dataset was then converted into a structured format on a spreadsheet file. A total of 14 variables were identified (13 input variables and 1 output/decision variable). The dataset consist of previous criminal and civil appeal cases and their judgment which were delivered by the SCN between 1962 and 2022. A total of 5585 appeal cases brought before the SCN were collected from the archive.
Data source location	• Institution: Supreme Court of Nigeria • City/Town/Region: Abuja • Country: Nigeria The primary dataset was provided by the Primsol Law Pavillion who owns an independent online archive of court cases which can be publicly accessed via subscription.
Data accessibility	Repository name: Mendeley doi:10.17632/ky6zfyf669.1 Direct URL to data: https://data.mendeley.com/datasets/ky6zfyf669/1

## 1 Value of the Data

• The dataset consist of information about approved and rejected appeal cases that were presented at the Supreme Court of Nigeria (SCN). The data can be useful in developing predictive systems which can provide effective decision support needed in facilitating the efficient delivery of appeal cases brought before the SCN.
• The data will prove useful to data scientist and machine learning enthusiast for the application of supervised and unsupervised machine learning algorithms which can reveal previously unseen patterns and relationships between identified variables.
• The dataset can be useful to lawyers and machine learning experts for the development of classification models which will aid in decisions affecting the outcome of appeal cases.
• Algorithmic decision predictors which are an important part of the dataset have the tendency to improve the predictability and consistency of judicial and decision making as demanded by the principle of equity.

## 2 Objective

The main goal of generating the dataset is to provide a means via which the judiciary process is improved in Nigeria. The information contained in the dataset can provide a means for assessing the underlying relationship that exists between the identified factors and the outcome of the judicial process. The data can be used to support the decision-making process which may affect the outcome of an appeal case brought before the SCN. The ability to determine the potential judgment of a case based on the information provided could help assist a lawyer in identifying the best possible strategy to be applied. The analysis of the data by lawyers can help in understating the underlying relationship that lies among variables and on the determination of the outcome of judgments. The utilization of the data by lawyers can reduce the time and money spent in searching through voluminous texts for the purpose of generating the exact and accurate information needed to understand the distribution of certain elements of court cases.

## 3 Data Description

Technology has played a vital role in creating a foundational basis for the adoption of artificial intelligence (AI) [1]. Introducing AI to the justice system promises to improve procedural and administrative efficiency, aid in decision making processes for judges, lawyers and litigants, and further predict outcomes consistent with past precedents [2], [3], [4]. [5,6] are few of the researchers who have explored this area.

The dataset named appeal_cases.csv consists of previous criminal and civil appeal cases and their judgment which were delivered by the Supreme Court of Nigeria between 1962 and 2022; a period of 60 years. The secondary dataset was provided by the Primsol Law Pavillion who owns an independent online archive of court cases which can be accessed via subscription. A total of 5585 appeal cases brought before the SCN were collected from the archive. The data collected from the archive consists of case files that were stored as text-based documents in the .docx format thus presenting the data as an unstructured dataset. The unstructured dataset was converted into a structured format and then presented on a spreadsheet file. The dataset consist of 14 variables that were painstakingly extracted hence giving it its unique and distinct nature.

Table 1 presents the various categories of the offence and respective sentence for each of the appeal cases. The table also shows the numerical value that was used to represent the various categories of offence and sentences presented in the collected dataset. Table 2 presents the categorization and transformation of the district of trail and appeal. The states reveal the state to which the cities belong to while the senatorial district is composed of a number of states. Each senatorial district was coded with an integer value that was used to replace the categorical values of each feature. Table 3 describes both the names of the dataset variables and what the values of the variables also mean.

**Table 1:** Categorization of offences and sentences.

Coded Value	Offence	Sentence
0	Wrongful accusations	Fine
1	Dispute (e.g., civil, land etc.)	Prison term
2	Court order violation	Prison term or Fine
3	Corruption/Abuse of office	Prison term with hard labour
4	Murder/Manslaughter	Suspension from Office
5	Libel	Death
6	Unlawful Possession	Payment for damages
7	Trespassing	Appeal granted
8	Civil Petition	Appeal dismissed
9	Theft/Break-in and entry	Other sentences
10	Unlawful termination of appointment	
11	Armed robbery	
12	Violation of human rights	
13	Rape/Sexual abuse	
14	Law of tort	
15	Election petition	
16	Conspiracy	
17	Claim for recovery/relief	
18	Fraud/Impersonation	
19	Damage of Property	
20	Other offences

**Table 2:** Categorization of district of trial and appeal.

States	Senatorial District	Integer Value
Benue, Kogi, Kwara, Nasarawa, Niger, and Plateau	North-Central	**1**
Adamawa, Bauchi, Borno, Gombe, Taraba, and Yobe	North-East	**2**
Jigawa, Kaduna, Kano, Katsina, Kebbi, Sokoto, and Zamfara	North-West	**3**
Akwa-Ibom, Bayelsa, Cross-River, Delta, Edo, and Rivers	South-South	**4**
Abia, Anambra, Ebonyi, Enugu, and Imo	South-East	**5**
Lagos, Ogun, Oyo, Osun, and Ogun	South-West	**6**
Federal Capital Territory		**7**

**Table 3:** Dataset description.

S/N	Variable Name	Variable Description	Remarks (all variables were stored using non-negative numeric values however the value -1 was used to identify missing values in the dataset)
**1**	**Appeal district**	the senatorial district of the appeal court;	Stored using the values 1 to 7; each representing the name of the senatorial district.
**2**	**Trial district**	the senatorial district of the trial court	Stored using the values 1 to 7; each representing the name of the senatorial district.
**3**	**Offence**	conducts or omissions that violate and are punishable under criminal law	Stored using the values 1 to 20; each representing the name of the offence.
**4**	**Sentence**	formal judgment of a convicted defendant in a criminal case setting the punishment to be meted out	Stored using the values 1 to 9; each representing the type of sentence.
**5**	**Number of complainants**	the number of person(s) that reports wrongdoing to law enforcement	Stored using a non-negative integer value, -1 represents missing values.
**6**	**Number of male complainants**	the number of male person(s) that reports wrongdoing to law enforcement	Stored using a non-negative integer value, -1 represents missing values.
**7**	**Number of female complainants**	the number of female person(s) that reports wrongdoing to law enforcement	Stored using a non-negative integer value, -1 represents missing values.
**8**	**Number of appeallants**	the number of persons who make an appeal to a higher court against a judgement made in a lower court	Stored using a non-negative integer value, -1 represents missing values.
**9**	**Number of male appeallants**	the number of male persons who make an appeal to a higher court against a judgement made in a lower court	Stored using a non-negative integer value, -1 represents missing values.
**10**	**Number of female appeallants**	the number of female persons who make an appeal to a higher court against a judgement made in a lower court	Stored using a non-negative integer value, -1 represents missing values.
**11**	**Number of public witness(es)**	the number of person(s) not a party and not called by a party to testify at a hearing	Stored using a non-negative integer value, -1 represents missing values.
**12**	**Number of eye witness(es)**	the number of person(s) who has seen something happen and can give a first-hand description of it	Stored using a non-negative integer value, -1 represents missing values.
**13**	**Number of defense witness(es)**	the number of witness(es) whom the appellant intends to call at a hearing or at trial	Stored using a non-negative integer value, -1 represents missing values.
**14**	**Final decision held (Judgment)**	the judgement made by the SCN regarding the appeal as either approved or disapproved	Stored using a non-negative integer value, -1 represents missing values.

Table 4 shows the result of the statistical distribution of the numeric features in the dataset based on a number of statistics. The analysis was done using descriptive statistical analysis of the values by estimating the mean, minimum, maximum, median, mode, standard error, standard deviation and other related statistic.

**Table 4:** Descriptive Statistics of the distribution of numeric varables.

Statistic	number of complainants	number of male complainants	number of female complainants	number of appellants	number of male appellants	number of female appellants	number of public witnesses	number of eye witnesses	number of defense witnesses
**Mean**	1	0	0	1	1	0	0	0	0
**Standard Error**	0.04	0.02	0.01	0.03	0.02	0.01	0.04	0.01	0.02
**Median**	1	0	0	1	1	0	0	0	0
**Mode**	1	0	0	1	1	0	0	0	0
**Standard Deviation**	1.80	0.63	0.36	1.93	1.00	0.30	2.00	0.79	0.91
**Sample Variance**	3.23	0.39	0.13	3.72	1.00	0.09	3.99	0.62	0.82
**Kurtosis**	226.89	6.11	19.94	193.28	22.50	21.87	22.79	101.53	170.24
**Skewness**	12.52	1.80	3.83	10.85	4.11	4.28	4.13	8.84	10.20
**Range**	41	5	4	46	10	3	22	14	23
**Minimum**	1	0	0	1	0	0	0	0	0
**Maximum**	41	5	4	46	10	3	22	14	23

## 4 Experimental Design, Materials and Methods

### 4.1 Data acquisition

Before developing the dataset, a thorough review of the literature covering various areas was initially conducted in order to have a good understanding of the underlying concepts. It was observed that every case consists of a number of components namely: the case identity number, date of the case, location of trial and appeal, information about the appellant, complainants, and witnesses, offence committed, sentence declared, determination of appeals, introduction to the appeal, facts covered, issues and the decision held by the judges regarding the appeal. The various features that were identified include the following:

i. Appeal district – the senatorial district of the appeal court;
ii. Trial district – the senatorial district of the trial court;
iii. Offence – conducts or omissions that violate and are punishable under criminal law;
iv. Sentence – formal judgment of a convicted defendant in a criminal case setting the punishment to be meted out.
v. Number of complainants - the number of person(s) that reports wrongdoing to law enforcement;
vi. Number of male complainants - the number of male person(s) that reports wrongdoing to law enforcement;
vii. Number of female complainants - the number of female person(s) that reports wrongdoing to law enforcement;
viii. Number of appellants – the number of persons who make an appeal to a higher court against a judgment made in a lower court;
ix. Number of male appellants - the number of male persons who make an appeal to a higher court against a judgment made in a lower court
x. Number of female appellants - the number of female persons who make an appeal to a higher court against a judgment made in a lower court
xi. Number of public witness(es) – the number of person(s) not a party and not called by a party to testify at a hearing;
xii. Number of eye witness(es) – the number of person(s) who has seen something happen and can give a first-hand description of it;
xiii. Number of defense witness(es) – the number of witness(es) whom the appellant intends to call at a hearing or at trial;
xiv. Final decision held – the judgment made by the SCN regarding the appeal as either approved or disapproved.
xv. Number of eye witness(es) – the number of person(s) who has seen something happen and can give a first-hand description of it;
xvi. Number of defense witness(es) – the number of witness(es) whom the appellant intends to call at a hearing or at trial;
xvii. Final decision held – the judgment made by the SCN regarding the appeal as either approved or disapproved.

The information extracted from literature review was validated by professional lawyers and subsequently, data containing information about the factors were collected.

**Fig. 1:**
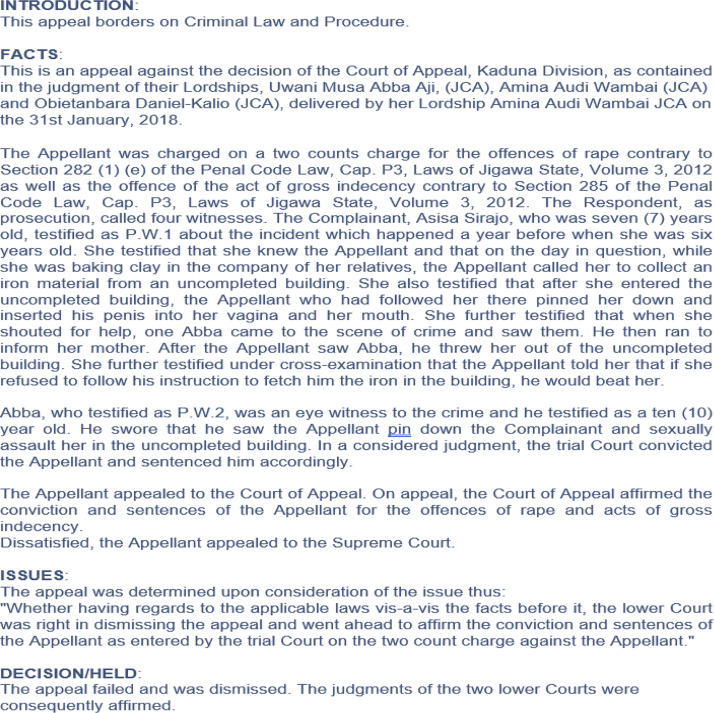
A sample of an appeal for a criminal case collected from the online archive.

**Fig. 2:**
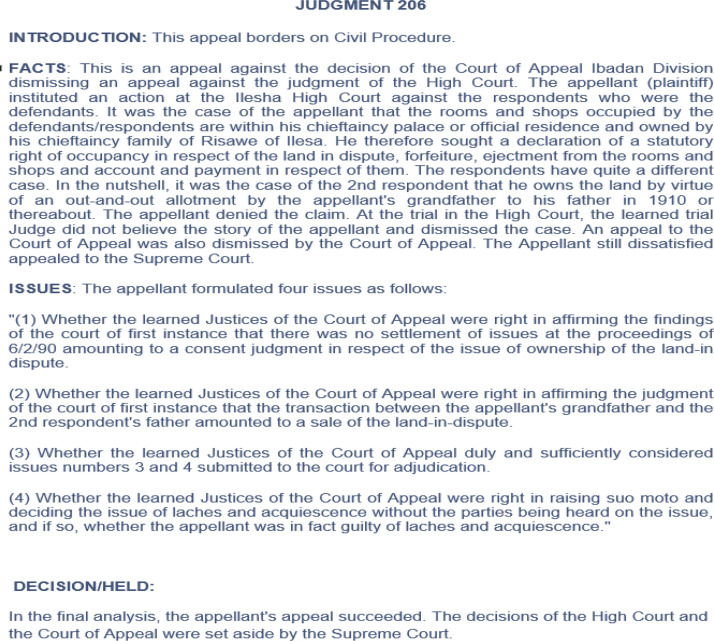
A sample of an appeal for a civil case collected from the online archive.

These variables were used as a basis for the extraction of the data that was stored in an online archive containing details about the outcomes of various appeal brought before the Supreme Court of Nigeria (SCN). The repository consists of electronic summaries of the proceedings of cases containing various sections such as introduction, facts, issues and the decision held by the SCN. Fig. 1, Fig. 2 show screenshots of the judgments of criminal and civil cases respectively brought before the SCN.

Each case has a serial number including district of appeal, district of trial, offence and sentence which were all captured as categorical values from the introduction, facts and issues components of the raw file. Also, information about the number of complainants, male complainants, female complainants, appellant, male appellant, public witness, eye witness and defense witness which were extracted from the fact's component of the raw file was sored as numeric values. Information about the outcome of each appeal according to the SCN was also collected and stored as a string variable called decision which contained the verdict of the SCN on every case. However, an additional variable was created named decision binary which was used to classify the decision based on granted and dismissed appeals alone. All other verdicts such as sustained, suspended, re-appeal were removed from the dataset alongside cases that were lost as a result of damages files.

The unstructured data stored in each text file was converted into a structured data containing information about a set of variables which were all extracted from documents collected from the online archive. Each part of the document was used to extract the information about each identified variable associated with the outcome of the cases while the decision held was used to determine if the appeal was either dismissed or granted. The city of the appeal and trial were classified according to their senatorial district in Nigeria to which their states belonged. This was done in order to reduce the set of values that were represented for each feature so as to reduce the complexity of the analysis of each feature. As a result of this, the city of trial and appeal were categorized into their respective senatorial districts. Each categorical variable was represented using a numeric value that lie between the numbers 1 and 7 as depicted in Table 2. Each senatorial district was coded with an integer value that was used to replace the categorical values of each feature.

More so, the various offences and sentence declared for each appeal case which was represented as categorical string values were converted into numeric values. More so, the target variable which contained information about the judgment of the appeal cases by the SCN was classified into two classes that were converted to binary values; 0 and 1 for the dismissed and granted appeal cases respectively.

### 4.2 Data pre-processing

**Fig. 3:**
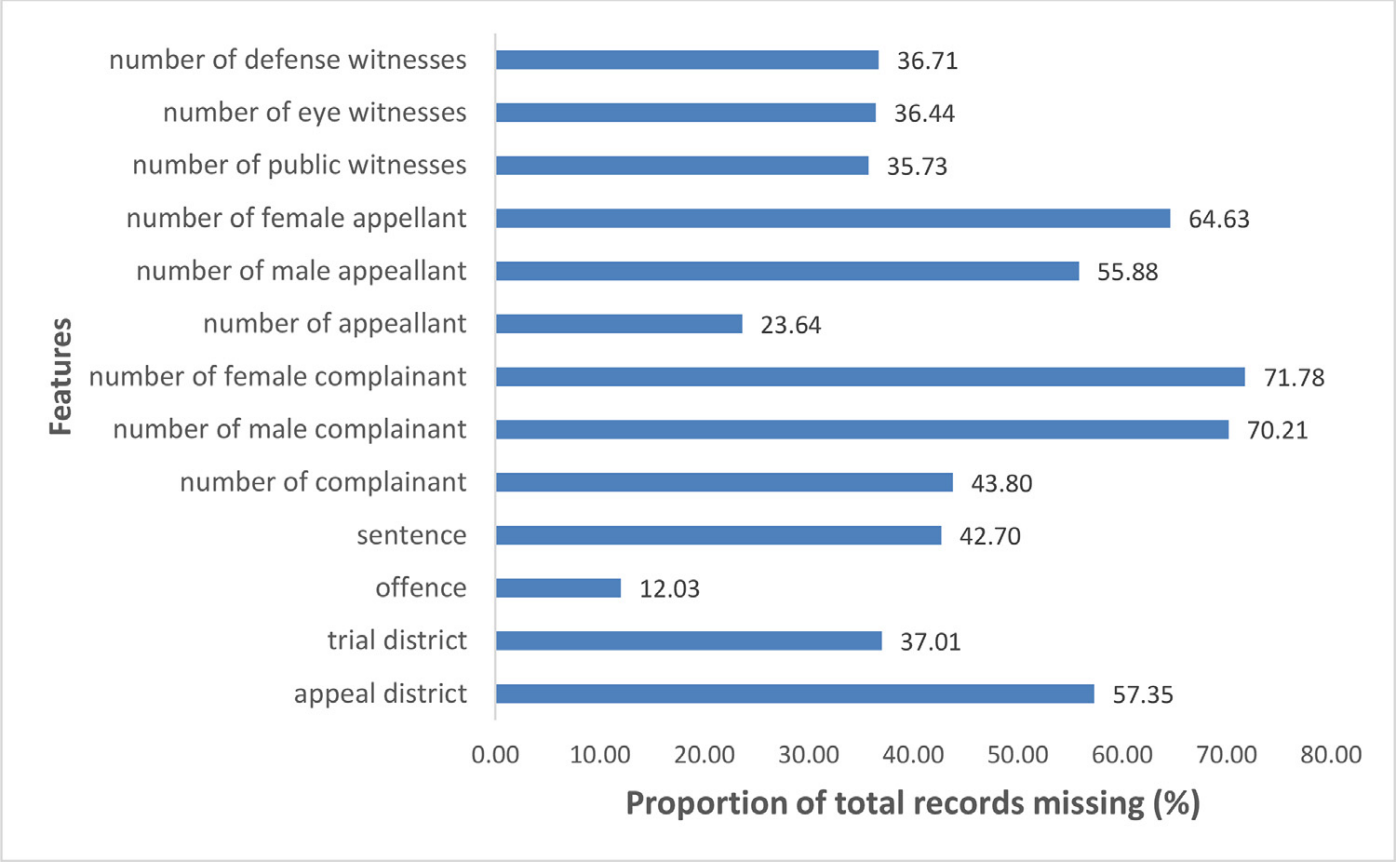
Distribution of missing values across features.

The pre-processing of the dataset was needed in order to eliminate the inconsistencies created as a result of noise in the dataset due to the presence of missing values across the features. Fig. 3 shows a description of the distribution of the proportion of the total records that were missing from the values of the features that were extracted from the case files in the dataset collected for this study. According to the figure, it was observed that as much as up to 72% of the total records were missing from the values of the number of male and female complainants however the values of the total number of complainants accounted for up to 44% of total records missing. Since all the features in the dataset had been converted to a numeric value, all the missing values within the feature set were replaced with a dummy value of -1. By doing this, subsequent missing values that are encountered within the dataset could be replaced with a dummy value thus making the dataset more suitable for use.

## Ethics Statement

The authors read and strictly adhered to all the ethical requirements for publication in Data in Brief. We confirm that that the current work does not involve human subjects, animal experiments or any data collected from social media platforms. The primary data used was gotten from a publicly available subscription based platform with permission and all records used were anonymized.

## CRediT authorship contribution statement

**O.C. Ngige:** Conceptualization, Methodology, Software, Data curation, Writing – original draft. **F.Y. Ayankoya:** Supervision, Software, Validation. **J.A. Balogun:** Conceptualization, Methodology, Software, Data curation, Writing – original draft, Visualization, Investigation, Software, Validation, Writing – review & editing. **E. Onuiri:** Conceptualization, Methodology, Software, Data curation, Writing – original draft. **C. Agbonkhese:** Software, Validation, Writing – review & editing. **F.A. Sanusi:** Visualization, Investigation, Supervision, Writing – review & editing.

## Declaration of Competing Interest

The authors declare that they have no known competing financial interests or personal relationships that could have appeared to influence the work reported in this paper.

## Data Availability

Appeal Cases heard at the Supreme Court of Nigeria Dataset (Original data) (Mendeley Data).

## References

[1] Jacob de Menezes-Neto E., Clementino M.B.M. (2022). Using deep learning to predict outcomes of legal appeals better than human experts: a study with data from Brazilian federal courts. PLoS ONE.

[2] Ogonjo F. (2021). https://cipit.strathmore.edu/ai-in-the-judicial-system-possible-uses-and-ethical-considerations/.

[3] R.W. Campbell, Artificial Intelligence in the Courtroom: The Delivery of Justice in the Age of Machine Learning (March 30, 2020). Ray Worthy Campbell, Artificial Intelligence in the Courtroom: The Delivery of Justice in the Age of Machine Learning, 18 COLO. TECH. L.J. 323 (2020), Peking University School of Transnational Law Research Paper, Available at SSRN: https://ssrn.com/abstract=4425791 or doi:10.2139/ssrn.4425791.

[4] J.G. Gogwim, Position of artificial intelligence in justice system: justice of the future, 2021 Retrieved from https://nji.gov.ng/wp-content/uploads/2021/12/Position-of-Artificial-Intelligence-in-Justice-System-Justice-of-the-Future-by-Joel-Gogwim.pdf.

[5] Medvedeva M., Wieling M., Vols M. (2023). Rethinking the field of automatic prediction of court decisions. Artif. Intell. Law.

[6] Bhilare P., Parab N., Soni N., Thakur B. (2019). Predicting outcome of judicial cases and analysis using machine learning. Int. Res. J. Eng. Technol..

